# Athletic retirement: factors contributing to sleep and mental health problems

**DOI:** 10.3389/fpsyg.2024.1350925

**Published:** 2024-03-15

**Authors:** Ashley Montero, John Baranoff, Robert Adams, Murray Drummond

**Affiliations:** ^1^College of Education, Psychology and Social Work, Flinders University, Bedford Park, SA, Australia; ^2^Sport, Health, Activity, Performance and Exercise (SHAPE) Research Centre, Flinders University, Bedford Park, SA, Australia; ^3^Flinders Health and Medical Research Institute (FHMRI) Sleep Health, Bedford Park, SA, Australia; ^4^School of Psychology, The University of Adelaide, Adelaide, SA, Australia

**Keywords:** sport, anxiety, depression, gender, recency of retirement, sleep disorders

## Abstract

**Introduction:**

The aim of this investigation was to determine which factors were associated with symptoms of sleep and mental health disorders in former athletes.

**Methods:**

Former athletes (N = 173, 50% women) who retired from any competition level within the last 20 years participated in an online survey. The survey consisted of the Athlete Sleep Screening Questionnaire (ASSQ), Personal Wellbeing Index-Adult (PWI-A), Center for Epidemiologic Studies Depression Scale-Revised (CESD-R), and Generalised Anxiety Disorder Assessment (GAD-7).

**Results:**

Binary logistic regressions revealed that both age (OR = 0.95 [95% CI:0.92, 0.99], *p* = 0.007) and gender (OR = 2.28 [95% CI:1.09, 4.79], *p* = 0.029) were associated with anxiety, with women and younger ex-athletes presenting greater risk of anxiety symptoms. Higher body mass was associated with an increased risk for sleep difficulty (OR = 1.13 [95% CI:1.03, 1.23], *p* = 0.008), sleep disordered breathing (OR = 1.20 [95% CI:1.10, 1.30], *p* < 0.001), and compromised wellbeing (OR = 0.89 [95% CI:0.83, 0.96], *p* = 0.001). Athletes who subjectively placed a lower priority on sport while competing presented greater risk of sleep disordered breathing (OR = 2.00[95% CI:1.05, 3.80], *p* = 0.035). No associations between recency retirement and any outcome measures were observed.

**Discussion:**

Findings suggest potential predictive factors for difficulty transitioning out of sport. Future longitudinal research should consider the interplay between sport re-engagement and the incidence and chronicity of sleep and mental health disorders.

## Introduction

Athletic retirement can be psychologically distressing, particularly when it is sudden and involuntary (Montero et al., 2022). Navigating this transition out of sport brings substantial lifestyle and social changes, which is a difficult time for many, especially those who highly identify with their athletic role (Park et al., 2013; Giannone et al., 2017) and those forced to retire (Esopenko et al., 2020). Involuntary retirement from sport can leave an athlete emotionally unprepared for this transition (Esopenko et al., 2020). With unexpected career-ending injuries, post-retirement planning is often not yet considered (Kaul, 2017). For professional athletes, career termination results in a loss of income; therefore, they are not only mentally, but potentially financially unprepared for a life outside of sport (Fortunato and Marchant, 1999). Exiting the high physical and psychological demands of sport on their own terms can be a form of relief for some (Jones and Denison, 2017). However, this transition can contribute to depression, anxiety, disordered eating, substance abuse, and suicide (Montero et al., 2022). Psychological distress and poor sleep go hand in hand; worry, stress, and rumination after retirement can manifest into sleep problems (Fernandes et al., 2019). The incidence of mental ill-health is believed to be the most prominent among recently retired athletes (<15 years; Oltmans et al., 2022). Individuals are likely to still be navigating this adjustment period of life outside of sport long after retirement.

A range of causal factors may contribute to the high rates of sleep and mental health disorders observed in retired athletes (Kerr et al., 2012, 2014; Gouttebarge et al., 2016; Luyster et al., 2017; Willer et al., 2018). Lindqvist et al. (2014) reported that suicide rates are 2–4 times higher for former male athletes than the general male population. Research on the health of retired athletes has identified key risk factors for mental health problems immediately following athletic retirement. Social breakdown, high and exclusive athletic identity, and involuntary or unplanned retirement can cause reduced wellbeing and psychological distress during the transition out of sport (Wippert and Wippert, 2010; Cosh et al., 2012a; Gouttebarge et al., 2015; Giannone et al., 2017). Athletes place significant emphasis on sport such that they often fail to engage in alternatives, hindering the establishment of their identity outside of sport (Hatamleh, 2013). In terms of gender, Lantz and Schroeder (1999) found that athletic identity is positively associated with masculinity and negatively associated with femininity in a cohort of male and female collegiate athletes. Indeed, men often identify more strongly with their athletic role than women (Brewer and Petitpas, 2017). These associations may be more prominent in sports that promote hegemonic masculine ideals such as stoicism, strength, and dominance (e.g., Australian football, rugby, cricket, etc.; Drummond et al., 2022). This may place males at greater risk of difficulty transitioning out of sport, as high athletic identity is associated with psychological distress following athletic retirement (Cosh et al., 2012a; Giannone et al., 2017). Furthermore, 70% of former male collegiate basketballers interviewed by Beamon (2012) reported a loss of personal and social identity after retirement, which led to identity crisis and depression. Australian skier Brittany George, discussed issues associated with suicide and mental illness during a recent podcast “Couching the Mind” (Woolaston, 2021). When reflecting on her life after retirement due to a back injury, she explained, “it has literally been my whole life, I’ve been ‘the athlete’ from when I was 2 until when I was 20 or 21,” “I did not have an identity. I was labelled ‘the athlete’ from a very young age and just rode with it” (Woolaston, 2021). Just months later, in January 2022, George took her own life.

A small body of research on the sleep health of retired athletes suggests a high prevalence of disordered sleep. Retired male National Football League (NFL) players have been a major focus of sleep research, demonstrating a high prevalence of sleep disordered breathing, being 2.5 times more likely to have sleep apnoea than a matched community cohort (Hyman et al., 2012; Luyster et al., 2017). For sleep disorders such as sleep apnoea, we understand that increased neck circumference and BMI are risk factors (Emsellem and Murtagh, 2005). Not only is this important for identifying potential risk profiles, but sleep apnoea has strong links with depression too (Kaufmann et al., 2017). Insomnia is extensively understudied in athletic populations, especially after retirement. A meta-analysis by Gouttebarge et al. (2019) identified that 20.9% of former athletes studied have sleep disturbances, suggesting symptomology of insomnia. Worryingly, insomnia is often comorbid to mental health disorders such as anxiety and depression (Khurshid, 2018).

Support networks and access to resources are typically removed after retirement (Jewett et al., 2019). This reduction in support not only thwarts the coping mechanisms required to deal with threats to mental ill-health and disordered sleep, but a lack of support prevents help-seeking and receiving necessary treatment. Inadequate support is problematic, as we know that the treatment of sleep disorders such as sleep apnoea have been efficacious in reducing symptomology and preventing mental health problems like depression (Zheng et al., 2019; Wimms et al., 2020). The removal of these support networks and consequential lack of treatment may inadvertently prevent the management of sleep health, perpetuating the cyclical nature of disordered sleep and mental ill-health, thus culminating to poor health outcomes.

Post-retirement research to date has focussed on predominantly elite competitors (Montero et al., 2022). Most research in this field has recruited athletes who compete at an elite/international level and world class level as defined by McKay et al.’s (2022) performance classification framework. Consequently, we have little knowledge of how retirement transitions affect athletes from other competition levels (i.e., highly trained/national level, trained/developmental, and recreationally active). Additionally, there is little knowledge about the sleep health of former athletes. Further exploration is warranted for factors that do not necessarily relate to the circumstances of retirement. Identifying risk factors which contribute to sleep and mental health problems is essential for early identification, prevention, and treatment.

Therefore, the aim of this study is to investigate whether factors such as age, gender, body mass, recency of retirement, and priority placed on sport are associated with the prevalence and severity of sleep and mental health problems following retirement from sport. In the same way that high athletic identity is associated with greater psychological distress following retirement (Cosh et al., 2012a; Giannone et al., 2017), investigating whether the priority level placed on sport while competing has any effect on psychological distress after retirement would be advantageous. A higher priority placed on sport (i.e., winning at all costs) is thought to relate to a greater emphasis on an athlete’s athletic role. We hypothesise that a higher priority level placed on sport whilst competing will be associated with greater sleep and mental health problems. With more emphasis placed on sport, the removal of this integral part of their life is thought to elicit significant psychological distress and in turn, produce sleep problems. Drawing from previous literature (Giannone et al., 2017; Oltmans et al., 2022), we hypothesise that more recently retired athletes are more vulnerable to sleep and mental health issues, whilst still adjusting to life outside of sport. Since men typically identify more strongly with their athletic role than women (Drummond, 2019), we hypothesise that they will have placed greater priority on sport during their careers and therefore be at greater risk for sleep and mental health problems following retirement. Since body mass is a predictor of sleep disordered breathing (Emsellem and Murtagh, 2005; Senaratna et al., 2016), which is associated with poorer mental health (Kaufmann et al., 2017), we hypothesise that increased body mass will be associated with greater sleep and mental health difficulties.

## Materials and methods

### Research design

A cross sectional prospective study design was used to assess data from a large scale online quantitative survey from which non-probability sampling was employed. This study was part of a larger study (*N* = 946) that investigated the prevalence and severity of sleep and mental health problems in current and retired athletes.

### Participants

Convenience sampling was used to obtain survey responses from an open anonymous online survey. The presented data includes only the one hundred and seventy-three former athletes (women = 87, men = 86) who completed the survey. These athletes had retired from sports competition within the last 20 years. Demographic and sporting history information can be seen in Table 1.

**TABLE 1:** Participant characteristics.

Characteristic	*n*	%
**Age (years)**		
18–29	73	42.2
30–39	32	18.5
40–49	41	23.7
50–59	13	7.5
≥60	14	8.1
**Body mass index (BMI)**		
Underweight (<18.50)	5	2.9
Healthy range (18.50–24.99)	70	40.5
Overweight (25–29.99)	60	34.7
Obese (30 ≤ )	38	22.0
**Sport**		
American football/gridiron	9	5.2
Athletics	6	3.5
Australian rules football	10	5.8
Basketball	4	2.3
Bowling	1	0.6
Cheerleading/dance	3	1.7
Climbing/mountaineering/orienteering	3	1.7
Combat sports (e.g., boxing, karate, judo, etc.)	6	3.5
Cricket	6	3.5
Cycling	4	2.3
Equestrian	1	0.6
Football/soccer	12	6.9
Golf	1	0.6
Gymnastics/trampoline	9	5.2
Hockey/ice hockey	2	1.2
Lacrosse	1	0.6
Netball	5	2.9
Paddle sports (e.g., canoeing, rowing, etc.)	7	4.0
Racquet sports (e.g., tennis, squash, etc.)	7	4.0
Rugby	26	15.0
Skating	1	0.6
Skipping	4	2.3
Surf life-saving/surfing	2	1.2
Swimming/diving	22	12.7
Triathlon	3	1.7
Volleyball	4	2.3
Water polo	4	2.3
Weightlifting/cross fit	10	5.8
**Level of competition**		
Amateur	79	45.7
Sports Institute or university/college	49	28.3
Semi-professional	26	15.0
Professional	19	11.0
**Country of residence**		
Australia	106	61.3
Canada	2	1.2
Ireland	5	2.9
New Zealand	11	6.4
South Africa	2	1.2
United Kingdom	9	5.2
United States of America	38	22.0
**Priority placed on sport**	**79**	**45.7**
Low	9	5.2
Medium	48	27.7
High	116	67.1
**Recency of retirement (years)**		
0–5	100	57.8
6–10	34	19.7
11–15	22	12.7
16–20	17	9.8

### Materials/instruments

Several measures were used which contain the necessary psychometric properties to screen for sleep and mental health problems in athletes. All measures chosen are relevant to athletic populations, with questions also being relevant for non-athletic populations; thus, each screening measure is considered suitable for retired athletes. Sleep disordered breathing and sleep difficulty were screened for using the Athlete Sleep Screening Questionnaire (ASSQ; Samuels et al., 2016) and is recommended by the International Olympic Committee (IOC) Mental Health Working Group (Gouttebarge et al., 2021). The Generalised Anxiety Disorder–7 (GAD-7; Spitzer et al., 2006) was used to screen for anxiety, which is also recommended by the IOC Mental Health Working Group. The Center for Epidemiologic Studies Depression Scale–Revised (CESD-R; Eaton et al., 2004) was chosen to screen for depression as it is based on more updated Diagnostic and Statistical Manual of Mental Disorders (DSM) criteria than the IOC Mental Health Working Group’s recommended depression screening measure. Currently, there is no consensus approach to measuring subjective wellbeing in athletic populations, therefore the Personal Wellbeing Index–Adult (PWI-A) was chosen due to its proven reliability in diverse populations (International Wellbeing Group, 2013). Participants were asked about their demographics, sporting history, and retirement circumstances.

### Procedure

The voluntary survey was available to adult athletes from all sports, genders, and levels of competition, at any stage of their career. Sporting organisations from Australia, Canada, Ireland, New Zealand, South Africa, United Kingdom, and United States of America were approached to promote the study. Further advertising was conducted via social media, recruitment flyers, and word of mouth (i.e., passive snowballing). All participants provided their informed consent online prior to completing the survey. This research was approved by the Flinders University Human Research Ethics Committee (Project ID 4276) and was conducted in accordance with the Declaration of Helsinki (World Medical Association, 2013) and Standards for Ethics in Sport and Exercise Science Research (Harriss et al., 2022).

### Statistical analysis

The independent variables of interest were age, body mass index (BMI), gender, recency of retirement [i.e., duration since retirement from competition (years)] and priority level placed on sport: low (i.e., maintaining health and fitness, played mainly for the social aspect); medium (i.e., competitive, desire to play at a reasonable level); or high (i.e., extremely competitive, desired to make representative/professional teams). Dependent variables from the screening tools were prevalence (i.e., based on cut-off scores) which was categorised into minimal risk or at risk (Table 2). Those who reported that they loudly snore, choke, gasp, or stop breathing during sleep were deemed at risk for sleep disordered breathing (ASSQ Sleep Disordered Breathing).

**TABLE 2:** Diagnostic criteria and risk thresholds for outcome measures.

	Minimal risk	At risk			
CESD-R (0–60)	No clinical significance: <16	Subthreshold depressive symptoms, but at risk: ≥16	Possible MD: 2 DSM symptoms for at least 5–7 days in past week*	Probable MD: 3 DSM symptoms for at least 5–7 days in past week*	Meets criteria for MD: 4 DSM symptoms nearly every day for past 2 weeks*
ASSQ Sleep Difficulty Score (SDS: 0–17)	None: 0–4	Mild: 5–7	Moderate: 8–10	Severe: 11–17	
GAD-7 (0–21)	Minimal: 0–4	Mild: 5–9	Moderate: 10–14	Severe: 15–21	
PWI-A (0–100)	Normal levels: ≥70	Compromised levels: ≤69			

*Plus anhedonia or dysphoria nearly every day for past 2 weeks.

IBM SPSS Statistics V28 (IBM Corp; Armonk, NY, USA) was used for statistical analysis of these data. Using cutoff scores for diagnostic criteria, binary logistic regressions revealed the odds of athletes presenting clinical disorders based on the recency of retirement (years), priority level (low, medium, high), gender (women, men), age (years), and BMI (kg/m^2^). An alpha level of 0.05, with 95% confidence intervals were used for all statistical tests.

Across all outcome measures, a total 4.62% of data was missing. A Little’s missing completely at random (MCAR; Little and Rubin, 2019) test revealed that data were missing completely at random (*p* = 0.351). The amount of missing data was below the consensus threshold of missingness (5%; Schafer, 1999) and the analyses were unlikely to be biased as a result (Bennett, 2001). Considering these factors, no imputation methods for missing data were used. Tests to see if the data met the assumption of collinearity indicated that multicollinearity was not a concern [gender, tolerance = 0.77, variance inflation factor (VIF) = 1.29; age, tolerance = 0.56, VIF = 1.79; BMI, tolerance = 0.80, VIF = 1.26; recency of retirement, tolerance = 0.70, VIF = 1.43; priority level, tolerance = 0.82, VIF = 1.22].

## Results

**FIGURE 1:**
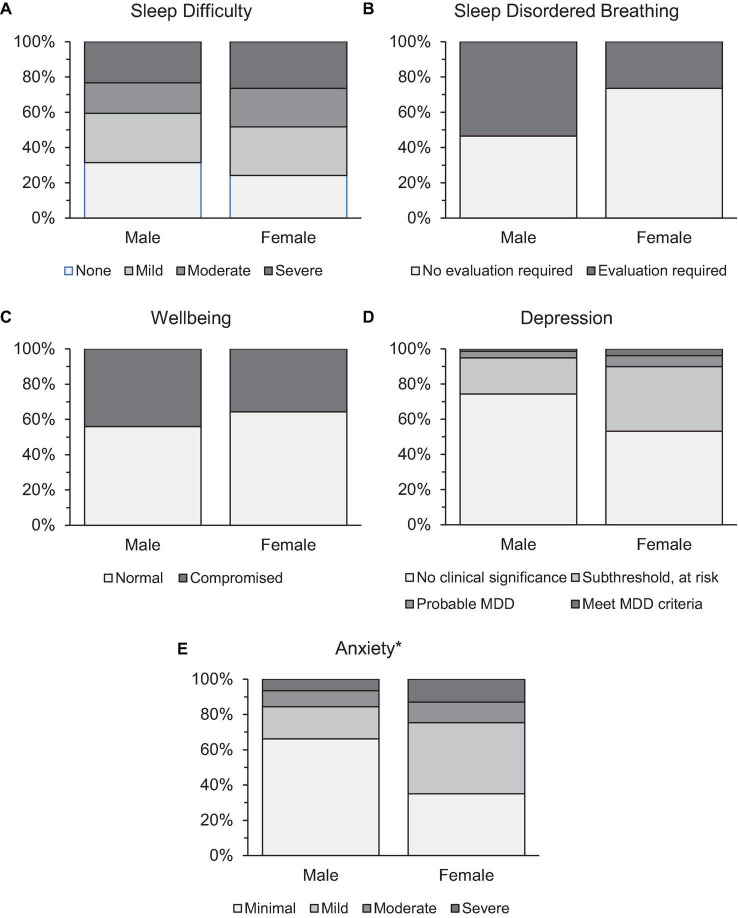
Prevalence rates of retired athletes at risk for panel **(A)** sleep difficulty, **(B)** sleep disordered breathing, **(C)** compromised wellbeing, **(D)** depression, and **(E)** anxiety by gender. *Significance at *p* < 0.05 level.

**FIGURE 2:**
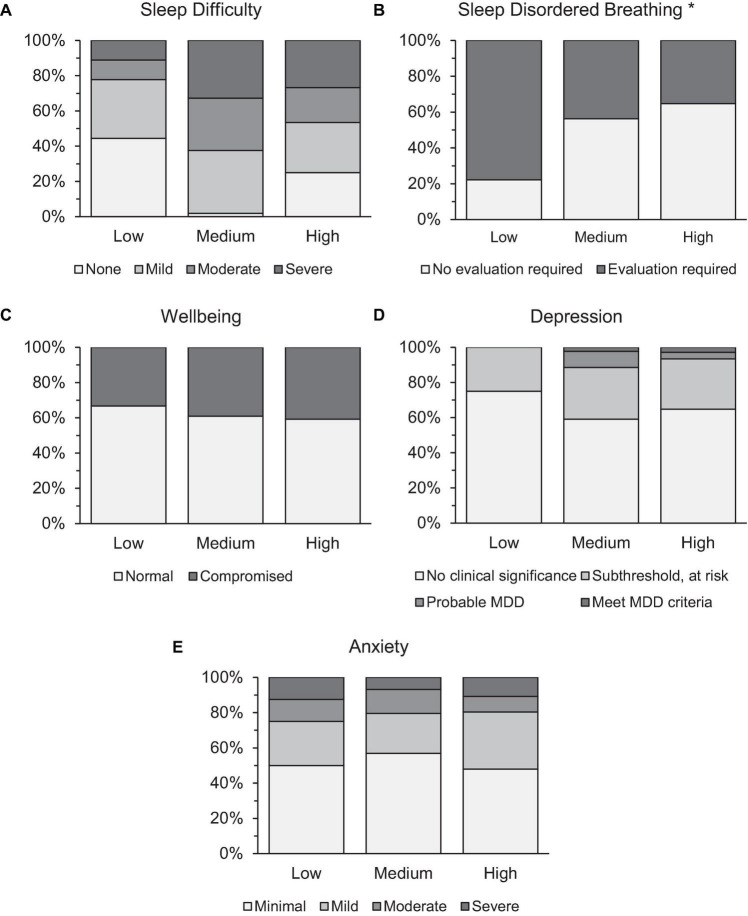
Prevalence rates of retired athletes at risk for panel **(A)** sleep difficulty, **(B)** sleep disordered breathing, **(C)** compromised wellbeing, **(D)** depression, and **(E)** anxiety by priority placed on sport during their career. *Significance at *p* < 0.05 level.

Former athletes from 28 different sport types, who retired from any level of competitive sport within the last 20 years were screened for point prevalence risk for sleep and mental health problems. Binary logistic regressions were performed to ascertain the effects of age, BMI, gender, recency of retirement, and priority level on the odds that participants were at risk for sleep and mental health problems. As seen in Table 3, models were statistically significant for predicting risk for sleep difficulty, sleep disordered breathing, anxiety, and wellbeing, but not depression (Figures 1D, 2D). For sleep difficulty, BMI predicted risk, but not age, gender (Figure 1A), priority placed on sport (Figure 2A), or recency of retirement; whereby higher body mass was associated with greater risk for sleep difficulty. For sleep disordered breathing, BMI and priority placed on sport predicted risk (Figure 2B), but not age, gender (Figure 1B), or recency of retirement; whereby higher body mass and lower priority placed on sport were associated with a greater risk of sleep disordered breathing. For compromised wellbeing, BMI predicted risk, but not age, gender (Figure 1C), priority placed on sport (Figure 2C), or recency of retirement; whereby higher body mass was associated with a greater risk of compromised wellbeing. For anxiety, both age and gender (Figure 1E) were associated with risk, but not BMI, priority placed on sport (Figure 2E), or recency of retirement; whereby younger individuals and women presented a greater risk of anxiety.

**TABLE 3:** Logistic regression model inferential statistics.

Model	OR	[95% CI]	*p*
ASSQ: sleep difficulty [X^2^ (5) = 11.79, p = 0.038*]			
Age (years)	0.98	[0.94, 1.01]	0.234
BMI (kg/m^2^)	1.13	[1.03, 1.23]	0.008*
Gender (men, women)	1.77	[0.80, 3.92]	0.161
Priority placed on sport (low, medium, high)	0.69	[0.37, 1.28]	0.238
Recency of retirement (years)	1.00	[0.92, 1.08]	0.945
ASSQ: sleep disordered breathing [X^2^ (5) = 42.16, *p* < 0.001*]			
Age (years)	0.98	[0.95, 1.02]	0.283
BMI (kg/m^2^)	1.20	[1.10, 1.30]	<0.001*
Gender (men, women)	0.47	[0.22, 1.02]	0.057
Priority placed on sport (low, medium, high)	2.00	[1.05, 3.80]	0.035*
Recency of retirement (years)	1.05	[0.97, 1.13]	0.219
PWI-A: wellbeing [X^2^ (5) = 14.87, p = 0.011*]			
Age (years)	1.03	[1.00, 1.07]	0.076
BMI (kg/m^2^)	0.89	[0.83, 0.96]	0.001*
Gender (men, women)	1.20	[0.57, 2.50]	0.636
Priority placed on sport (low, medium, high)	1.04	[0.56, 1.92]	0.899
Recency of retirement (years)	0.96	[0.90, 1.03]	0.297
**CESD-R: depression [X^2^ (5) = 8.98, p = 0.110]**			
Age (years)	0.98	[0.95, 1.02]	0.326
BMI (kg/m^2^)	1.00	[0.93, 1.07]	0.982
Gender (men, women)	2.27	[1.07, 4.80]	0.032*
Priority placed on sport (low, medium, high)	1.22	[0.66, 2.28]	0.529
Recency of retirement (years)	1.03	[0.96, 1.12]	0.216
GAD-7: anxiety [X^2^ (5) = 25.44, *p* < 0.001*]			
Age (years)	0.95	[0.92, 0.99]	0.007*
BMI (kg/m^2^)	0.97	[0.91, 1.04]	0.432
Gender (men, women)	2.28	[1.09, 4.79]	0.029*
Priority placed on sport (low, medium, high)	1.17	[0.60, 2.26]	0.647
Recency of retirement (years)	1.06	[0.98, 1.14]	0.182

*Denotes significance at the *p* < 0.05 level (two-tailed).

## Discussion

Extending previous research investigating predictors of difficulty following athletic retirement (i.e., forced/involuntary retirement, high athletic identity), we explored factors such as age, gender, body mass, recency of retirement, and perceived priority placed on sport. All factors except recency of retirement predicted risk for some form of sleep or mental health issue; although sleep and mental health problems may have been present whilst competing, findings provide some indication of predictive factors for presenting symptomology following retirement from sport.

Oltmans et al. (2022) found that retired athletes are most susceptible to psychological distress during the first 15 years of retirement. However, in this sample, recency of retirement was not associated with the risk of presenting symptomology for sleep or mental health problems, despite the assumption that perceived adjustment to athletic transitions generally improves over time (Reifsteck et al., 2023). With 90.2% of the sample having completed the survey within 15 years of retiring from competition, perhaps sleep and mental health issues are highly prevalent and severity does not necessarily differ across time due to the nuanced and varied experiences of individuals (Stambulova et al., 2021).

Extensive literature suggests that high and exclusive athletic identity predicts psychological distress following retirement from sport (Park et al., 2013; Giannone et al., 2017). Priority placed on sport while competing may be linked to athletic identity. However, we found that the priority placed on sport did not predict risk for depression, anxiety, compromised wellbeing, or sleep difficulty. Those who placed a low priority on sport while competing were at greater risk to sleep disordered breathing. One possible explanation is that this low priority of sport during competition may have reflected a lower priority on physical activity and healthy eating, which has endured following retirement. This may be related to body mass; we know that increased body mass is associated with sleep disordered breathing (Emsellem and Murtagh, 2005; Senaratna et al., 2016), which was also observed in this sample.

Body mass also predicted risk for sleep difficulty and compromised wellbeing. A study of ultra-marathon swimmers found that higher BMI was associated with reduced total sleep time (Dunican et al., 2022). A systematic review and meta-analysis by Amiri (2023) found a direct linear relationship between BMI and sleep disturbances. Potential mechanisms identified for this association were an accumulation of fat in the respiratory tract, in the chest and in the abdomen that causes respiratory issues, and subsequently disturbed and fragmented sleep (Amiri, 2023). From a psychological standpoint, the association between increased body mass and compromised wellbeing may reflect lower satisfaction with life domains such as health, self-esteem, achievement, and standard of living (Wardle and Cooke, 2005; Morris et al., 2010; Haga et al., 2019). These findings highlight the importance of maintaining healthy habits following the cessation of sport.

Abel and Carreiro (2011) suggest that sport science professionals (e.g., strength and conditioning coaches, dietitians, etc.) should educate athletes and help them to develop the skills necessary for living a healthy lifestyle after sport. Evidence-based, theory-driven programmes that assist with developing goal-setting strategies have shown to improve motivation for engaging in physical activity and healthy eating for life after retirement (Reifsteck et al., 2018; Davis Brooks et al., 2019). Greater emphasis on education and awareness on recommended physical activity levels and nutrition guidelines may help to mitigate the association between body mass, poor sleep, and poor wellbeing.

We explored gender as a predictive factor for disordered sleep and mental ill-health following athletic retirement. Research suggests that males often have higher athletic identity (Sturm et al., 2011) and high athletic identity predicts psychological distress after retirement (Park et al., 2013; Giannone et al., 2017). Contrary to our hypothesis, retired women athletes had higher depression and anxiety scores than retired men athletes. Women had 2.28 times greater odds of presenting symptoms of anxiety. Interestingly, gender was not associated with disordered sleep. This may be attributed to the fact that there are mixed findings in relation to sex differences in athletes sleep quality (e.g., Carter et al., 2020; Kawasaki et al., 2020; Hrozanova et al., 2021; Vlahoyiannis et al., 2021; Romdhani et al., 2022), or potentially the fact that these sex differences are less pronounced following the cessation of sport. Therefore, findings suggest that retired women may experience greater psychological distress, but not poorer sleep. Similar trends have been observed in current athlete cohorts. Mascaro et al. (2021) reported that female athletes had high depression, anxiety, stress, and later sleep times than male athletes. An avenue of exploration would be gender differences in the reasons for retirement. For example, some women may be forced to retire from sport for family reasons, such as having children. While women appear to present greater risk for mental ill-health, research by DeFreese et al. (2021) found that social support and mental, nutritional, and physical health resources were catalysts for successful retirement transitions in former women’s soccer athletes—suggesting that appropriate support may mitigate these effects.

Like gender, age was associated with anxiety, whereby younger athletes were more anxious. Compared with other careers, athletes typically retire at a young age, therefore, they need to possess alternative skills and passions outside of sport, to move into other employment and career pathways (Cosh et al., 2012a). Initial transitions out of sport at an early age may be challenging as they navigate alternatives outside of sport, translating into increased levels of anxiety.

Several limitations should be addressed. Research suggests that those who have not planned for retirement face significant difficulty upon exiting sport (Knights et al., 2019). However, we did not obtain data regarding the level of preparation or planning for retirement. Future studies could simply employ a visual analog scale from 0 (not at all prepared) to 100 (extremely prepared), which may help to determine whether preparedness mediates difficulty following athletic retirement. Similarly, data was not obtained on the circumstances of retirement which are known to cause psychological distress (e.g., injury, contract termination; Kaul, 2017; Esopenko et al., 2020); investigation into the cause of retirement may indicate whether these factors mediate the effects observed in the present study.

To reduce survey length, data on physical activity, dietary behaviours, alcohol consumption, and drug use were not recorded. We observed that increased body mass was associated with difficulty following retirement and suspect that this is a consequence of deregulation of these behaviours. Further inquiry into these behaviours following retirement may uncover the mechanistic properties for poor sleep and mental ill-health after the cessation of sport.

Future research into fluid transitions and re-engagement back into sport, investigated by Cosh et al. (2012b), would be worthwhile. Some athletes return to sport after a period of retirement; it would be interesting to see how these different transition pathways affect sleep and mental health. Similarly, it would be valuable to investigate social integration in sporting clubs following retirement. This is perhaps more difficult at an elite level where there are often specialised roles. But at a community level, continued social connectivity or participation at a lower level has shown many health and social benefits (Jenkin et al., 2021). Longitudinal research would provide opportunity to monitor the contribution of developing mental health problems throughout and following athletes’ careers—an area Ivanović and Macak (2020) identified as being unexplored.

Evaluation and optimisation of support interventions surrounding retirement transitions would be beneficial. Programmes focussed on education and dialogue around retirement, developing coaching strategies, identifying resources, setting goals, and peer engagement have shown to enhance the transition experience (Hansen et al., 2019; Reifsteck et al., 2023). Investigation into the efficacy of sleep interventions following athletic retirement, as a means of both improving not only sleep but also mental health would also be advantageous. A meta-analysis by Gwyther et al. (2022) identified that sleep interventions in current athletes have shown to significantly improve negative affect. The authors highlighted the need for further research into the efficacy of sleep interventions for mental health improvement and prevention in athletic populations. Developing intervention frameworks combining elements of education around sleep, mental health, physical activity, nutrition, and help seeking within the context of athlete career transitions is viable and could facilitate healthier exits from sport.

Majority of participants were Australian (61.3%) and demographics are largely consistent with WEIRD criteria (i.e., Western, educated, industrialised, rich and democratic); thus, interpretations cannot be systematically generalised to all athletes across the globe. Nevertheless, this research provides preliminary evidence to suggest that factors such as age, body mass, gender, and priority level placed on sport predict risk of disordered sleep and mental ill-health following athletic retirement. These contributions appear to be more prominent for mental health domains such as anxiety and poor quality of life, than for sleep difficulty, sleep disordered breathing and depression. There is no evidence to indicate that recency of retirement is associated with these issues in the 20 years following retirement from sport. Although we have identified potential factors associated with the risk for sleep and mental health issues following athletic retirement, we recognise that transition outcomes are not binary (i.e., adaptive or maladaptive; Stambulova et al., 2021), and those considered to have a “successful” transition journey are certainly not exempt from poor mental health and sleep difficulties.

Longitudinal research into the timing and chronicity of sleep and mental health issues would provide greater insight into predictive and contributing factors during and after an athletic career. By extending our current understanding of potential risk factors and profiles for mental ill-health and disordered sleep, sports organisations can better identify and mitigate these risks by tailoring support accordingly. While we endorse the implementation of education and support on health optimisation during an athlete’s sporting career, we argue that equal, if not greater, priority should be placed on support throughout retirement transitions. Adopting career transition interventions, physical activity and nutrition promotion strategies, and continued dialogue may facilitate easier transitions out of sport. Exploring the potential of employing sleep interventions as a preventative measure and management tool for improving mental health following retirement shows promise. In all, multidisciplinary efforts are needed by researchers to identify and optimise evidence-based interventions. Meanwhile, sport science professionals are needed to implement and tailor these support practices in a more holistic, athlete centred approach.

## Data availability statement

The data that support the findings of this study are available from the corresponding author upon reasonable request.

## Ethics statement

The studies involving humans were approved by the Flinders University Human Research Ethics Committee (Project ID 4276). The studies were conducted in accordance with the local legislation and institutional requirements. The participants provided their written informed consent to participate in this study.

## Author contributions

AM: Writing – review and editing, Writing – original draft, Visualization, Project administration, Methodology, Investigation, Formal Analysis, Data curation, Conceptualization. JB: Writing – review and editing, Supervision. RA: Writing – review and editing, Supervision. MD: Writing – review and editing, Supervision.
